# PRO-based follow-up as a means of self-management support – an interpretive description of the patient perspective

**DOI:** 10.1186/s41687-018-0067-0

**Published:** 2018-09-05

**Authors:** Caroline Trillingsgaard Mejdahl, Liv Marit Valen Schougaard, Niels Henrik Hjollund, Erik Riiskjær, Sally Thorne, Kirsten Lomborg

**Affiliations:** 1 Department of Public Health, Aarhus University, Bartholins Allé 2, DK-8000 Aarhus, Denmark; 2 The Research Program in Patient Involvement, Aarhus University Hospital, Palle Juul-Jensens Boulevard 99, DK-8200 Aarhus, Denmark; 3 WestChronic, Occupational Medicine, University Research Clinic, Aarhus University, Herning, Gl Landevej 61, DK-7400 Herning, Denmark; 4 Department of Clinical Epidemiology, Aarhus University Hospital, Olof Palmes Allé 43-45. DK-8200 Aarhus N, Aarhus, Denmark; 5 DEFACTUM, Social & Health Services and Labour Market, Central Denmark Region, Olof Palmes Allé 15, DK-8200 Aarhus, Denmark; 6 University of British Columbia School of Nursing, 2211 Wesbrook Mall, Vancouver, BC V6T 2B5 Canada; 7 Department of Clinical Medicine, Aarhus University, Palle Juul-Jensens Boulevard 82, DK-8200 Aarhus, Denmark

## Abstract

**Background:**

There is an increasing focus on the use of patient-reported outcome (PRO) measures to improve the quality and effectiveness of health care. PRO-based follow-up is a new model of service delivery, where the patient’s PRO measures are used as the very basis for outpatient follow-up.

**Objectives:**

This study aimed to explore how patients with epilepsy experience the use of PRO-based follow-up in three outpatient clinics in the Central Denmark Region. We also sought to explain how these experiences relate to self-management.

**Methods:**

Interpretive description was the methodological approach. We conducted in-depth individual interviews with 29 patients referred to PRO-based follow-up, each of whom had completed at least two PRO questionnaires. Participants were sampled based on purposive and theoretical sampling.

**Results:**

PRO-based follow-up may support patients’ self-management by a) increasing awareness of psychosocial problems, b) improving communication, c) increasing understanding of symptoms, d) facilitating change in health behavior and e) strengthening autonomy. Inhibitors for PRO measures as a means of self-management support were identified as a) feelings of rejection and disconnection, b) incomprehension of purpose of PRO-based follow-up, c) PRO measures being too standardized and negative and d) lack of confidence in own ability to assess PRO questionnaires.

**Conclusion:**

The findings demonstrate broad variation in the influences of PRO measures on patient’s self-management in life with epilepsy. Sense of ownership may explain this variation. We suggest supplementary clinical initiatives in order to enhance the benefits from PRO-based follow-up, particularly on how patients are allocated to this health care service.

**Electronic supplementary material:**

The online version of this article (10.1186/s41687-018-0067-0) contains supplementary material, which is available to authorized users.

## Background

There is an increasing focus on the use of patient-reported outcome (PRO) measures to improve the quality and effectiveness of health care [1]. PRO is a measurement based on a report that comes directly from the patient about the status of that patient’s health condition [2]. In Denmark, since 2016, PRO-systems are being implemented on a large scale, based on national initiatives [3]. However, we know little about what happens when ideas on a macro level hit the clinical practice arena [4].

What we know is that PROs have potential for allowing patients to actively participate in their own care [5–7]. Studies indicate that management of symptoms may be improved by the use of PRO measures in clinical practice and increase symptom-related actions by both patients and clinicians [8, 9]. A number of studies suggest that the use of PRO measures in clinical practice can improve patient-clinician communication, given that PRO data can be an incentive to patient-centred communication [10–14]. A systematic review of the impact of routine collection of PRO data found strong evidence that well-implemented PRO measures improved patient-provider communication and patient satisfaction but found only weak evidence for the impact of PRO measures on changes regarding patients’ self- management, health outcomes and health behavior [10].

The patient perspective has received little consideration when it comes to PRO measures in routine clinical practice and as a means of self-management support. In spite of the promising benefits, research has also emphasized that PRO measures cannot be considered a self-acting mechanism that necessarily leads to patient involvement [7, 15]. Specifically, a systematic review of the experiences of professionals’ use of PRO data to improve the quality of health care documented barriers to implementing PRO measures such as skepticism about the validity of patient self-reported data and preferences for physiological measurements [6]. Thus, a series of conditions and mechanisms may influence the use of PRO measures in clinical practice as a method for self-management support.

Self-management is defined as “the individual’s ability to manage symptoms, treatment, physical and psychosocial consequences and life style changes inherent in life with long term conditions” [16]. As the lives of patients with epilepsy not only include endurance of a predisposition to generate epileptic seizures but also neurobiological, cognitive, psychological and social consequences of this condition [17], we considered epilepsy to be an appropriate case exemplar for this study.

We aimed at exploring how patients with epilepsy experience PRO-based follow-up used in three outpatient clinics in the Central Denmark Region, where PRO data is used as the basis for demand driven outpatient follow-up. We also sought to explain how these experiences related to self-management in PRO-based follow-up.

## Methods

### Interpretive description

We chose Interpretive Description (ID) [18] as the methodological approach. ID is an applied, inductive research strategy stressing the importance of conducting research arising from and with the aim of improving clinical practice [18]. ID differs from other methodologies as it draws on elements derived from phenomenology, grounded theory and ethnography but refrains from formalizing specific techniques and procedures as ultimate standards and goals of research [18]. Thus, ID does not prescribe an exact way to carry out the study, but rather represents an operating logic within which qualitative studies can be designed and enacted [18]. In accordance with ID we gathered and analyzed data concurrently, and thus let the preliminary data analysis guide the subsequent data collection. Preliminary analyses were noted in an audit trail throughout the research process to provide transparency.

### Setting

AmbuFlex is a generic web-administered PRO-system which aims to improve quality of care, increase the patient-centeredness of care, and reallocate health care resources by using PRO measures as the basis for follow-up [19, 20]. This is termed PRO-based follow-up. Thus, AmbuFlex differs from the traditional use of PRO measures in clinical practice, where PRO measures often just compose a supplement to the patient’s follow-up. PRO-based follow-up represents a new model of service delivery where the patient’s PRO measures are used as the very basis for outpatient follow-up. In PRO-based follow-up, regularly scheduled follow-ups are substituted with regular diagnosis-specific questionnaires filled in by the patient at home. The patients’ PRO-data are used by clinicians as a decision aid to identify those who need clinical attention based on an automated PRO-algorithm [21]. The patients can in all cases request a contact and thereby overrule any automated decision of no visit needed.

AmbuFlex/Epilepsy was implemented in 2012 in three different hospitals in The Central Denmark Region, and is standard follow-up for approximately 70% of all epilepsy outpatients in these clinics [20]. As of December 2017, 5181 epilepsy outpatients have been referred to AmbuFlex/Epilepsy, based on a clinical assessment of the patient’s health status and their ability to fill in questionnaires. The questionnaire encompasses information about health-related quality of life, seizures, other symptoms, information specific to aspects of daily life with epilepsy and the patient’s perception of well-being (Additional file 1). At the time of the data collection patients were not able to access their PRO measures once they had submitted their response, thus the patient’s PRO measures were only fed back to the clinicians.

### Sampling and data collection

Data collection was conducted by the first author and consisted of in-depth semi-structured interviews with 29 patients (Table 1). Interviews were conducted from December 2015 to June 2017 and were carried out in the patients’ home, except three interviews which, on patients’ requests, took place at the hospital. The inclusion criteria were patients referred to AmbuFlex/Epilepsy who had completed at least two PRO questionnaires. Using general questions and prompts designed to release patient narratives, participants were invited to share their personal experiences of AmbuFlex. The sampling was planned as purposive. Prior to the interviews we aimed to include patients representing a diversity in duration of epilepsy, gender and age. In accordance with the ID strategy of maximal variation associated with an evolving analysis, we supplemented our purposive sampling strategy with theoretical sampling [18]. Thus, when hunches about patterns and themes emerged during data collection and analysis, we sought to include specific types of participants to check if what we saw was an artifact of some unexplained characteristics of our current sample. For example, in the preliminary analysis we found great differences in patients’ experiences of feeling safe or abandoned in PRO-based follow-up. To be sure that these experiences could not be ascribed to patients’ established relation (or the lack of such) with the clinicians, we sampled participants with no or only minimal follow-up experiences prior to their enrolment into PRO-based follow-up. A diversity of experiences related to feeling safe or abandoned was identified amongst these participants, thus theoretical sampling helped us refine our claim about contrasting experiences. Although we had initially anticipated that a sample of 15–20 participants would be sufficient to yield thematic patterns within the dataset, the various nature of the documented experiences led us to continue recruitment. After 29 interviews had been completed, we concluded that sufficient common aspects were apparent in the dataset and the thematic patterns were sufficiently substantial to permit a comprehensive conceptual description of the whole.

**Table 1:** Participant profile

	*N* = 29 (%)
Gender	
Female	15 (52)
Male	14 (48)
Age	
20–35	10 (34)
36–50	4 (14)
51–65	7 (24)
> 65	8 (28)
Duration of epilepsy (years)	
< 5	2 (7)
6–15	9 (31)
16–30	10 (35)
31–45	5 (17)
> 45	3 (10)
Cohabitation	
Living with a partner	24 (83)
Living alone	5 (17)
Occupational status	
Working	16 (55)
Not working	13 (45)

### Analysis

All interviews were audio-recorded and transcribed verbatim by the first author. Data management was facilitated by the qualitative software program NVivo™ [22]. In accordance with ID concurrent data collection and analysis were performed [18]. Thus, preliminary analysis of the data influenced the consequent data collection. For example, an initial analysis revealed signs that some patients made different lifestyle choices due to PRO-based follow-up. Thus, a focus on PRO measures’ influence on health behavior was incorporated into the interview guide.

The first author conducted the initial rounds of analysis identifying patterns and subsequently themes, which were discussed and adjusted in close cooperation with the last author supported by discussions with co-authors. Once all data was gathered we conducted the final analysis with the first author being responsible of digging into the full data material. After carefully having read all the transcripts we organized data segments together that seemed to reflect similar properties, increasingly arranging the data in terms of patterns. Next, more formal coding was conducted, starting with a broad-based coding in order to avoid premature interpretations. As our data was organized in groups that might be thematically related, the specific dimensions of patients’ experiences were increasingly clustered into recurring themes. These tentative groupings allowed us to consider the patterns and variety within those groups across the whole material. As the analysis process developed and possible relationships between the groups of data became more apparent, we could finally conceptualize the findings by extracting thematic patterns that represent the potential of PRO-based follow up as a means of self-management support.

## Results

Potential participants were identified and approached by nurses from the three outpatient clinics and all agreed to participant in the interview, thus the final sample consisted of the 29 contacted patients (Table 1).

In general, our analysis revealed diverging attitudes toward PRO-based follow-up. For some patients, PRO-based follow-up was perceived as a quality improvement of the outpatient service. In particular, they valued the increased influence they could have on their follow-up. For other patients, PRO-based follow-up was experienced as a deterioration of the outpatient service. We found considerable variation in the influence of PRO-based follow-up on capacity for self-management. For some patients, signs that PRO measures were increasing their self-management capacity were clear. For others, we found equally clear indications that the procedure was impeding their follow-up experience and contact with the outpatient clinic. These contrasting experiences could explain both the supportive and inhibiting mechanisms relative to self-management. In representing experiences from opposite sides of a spectrum that covers significant variation in the documented experiences regarding PRO-based follow-up as a means of self-management support, the observed complexities can be illuminated. Illustrative quotations appear in Table 2.

**Table 2:** Examples of participants’ quotes

Supporting mechanisms	
Increasing awareness of psychosocial problems	At least the questionnaire is more profound than the usual how-are-you-questions. It seems as if they take you a bit more seriously now than they did before (68 years old man).
Improving communication	Well, I think it is very nice. The questions are much more everyday questions. That makes it much easier to explain and describe how your epilepsy actually is. Because you try and you try to explain how it is and how it feels to your close ones and to the doctors, but it is so hard to explain in a way that normal people can imagine how your body experiences it (28 year old woman).
Increasing understanding of symptoms and disease	It gets you thinking. It makes you consider things. Because I actually didn’t know that you could lose your sexual drive because of the medicine and that stuff. I surely didn’t know that before I saw the questionnaire (56 year old woman). Before I start to fill it in, I stop and think carefully about why the questions are there in the first place. They must be linked to the epilepsy. It isn’t like you have to find five errors [as in an intelligence test] or something like that. So, all the questions must have something to do with the epilepsy, right? And that makes you aware of symptoms that you must be attentive to (60 year old man).
Facilitating change in health behavior	There are things you do that you don’t link to the epilepsy by yourselves, but they can actually have an influence. So, in that way it can actually kind of guide you. [For example], maybe I should try and sleep a bit longer (28 year old woman). I actually think that just filling in the questionnaire and just by ticking those boxes made me more conscious, and then I said to myself “okay now I need to take on responsibility, because it is my life.” (48 year old man).
Strengthening autonomy, flexibility and freedom	I really think it is very very good alternative. I especially appreciate the fact that I actually get to control it myself (…). I really like the part that I can control my follow-up by saying “Do you know what? Now I need help.” (24 year old woman). I actually think it is all right [the fact that there is no routine visit anymore]. Because, I really don’t feel that I suffer from epilepsy. I actually don’t feel like that. I feel well in my everyday life, so I kind of forget that I have epilepsy. And sometimes I try to tell myself, that it is just something that they have made up. I don’t like the epilepsy, and this makes me feel less ill (66 year old man).
Inhibitors for self-management	
Inducing feelings of rejection and disconnection	When you write that you have lost your libido and you have gained weight, then what happens? Well, if I had been sitting in front of the nurse, then I would expect that she maybe could help me in some way (…) But if I just write it in the questionnaire, then what happens? – Nothing. Because I have never heard anything back, and I have been writing these things time after time (56 year old woman). I demand to know how my disease is developing! I want to know, is it okay? I would also like to have the possibility to ask some questions (…) I would really like to have the opportunity to tell how I feel and how I experience the side effects, and I don’t think that I have those opportunities now (23 year old woman). They never [expletive] react to it. So, I wonder, why do they ask about it in the first place? Honestly, why do they ask? Well, they don’t care about it at all. The only thing they react to is the medicine stuff. Why the [expletive] should I fill it in, then? It is nothing but a waste of time (58 year old woman). But I really think they ought to react to it - to follow up on the problems. Otherwise it is just like you disappear (47 year old woman). You somehow feel that you are abandoned from the system when you only have this [lays his hand on the questionnaire] (60 year old man).
Incomprehension of purpose of PRO-based follow-up	It is a survey. (…) I see it as some kind of data collection, a method for some nurses and doctors to become smarter on the disease (63 year old man). Well, they can’t use my part in itself. I think it is more in general that they use it. To see if people who take these medicines and who have epilepsy, to see if they have a direction, I think. So, I don’t think, that they use exactly my questionnaire, except as one in many (32 year old man).
PRO measures are perceived as being too standardized and negative	But I really don’t think all those things have anything to do with how I have epilepsy (23 year old woman). There are questions or areas that I personally think the questionnaire is lacking (…) In that way it can be difficult to fill it in, because I don’t think it fits 100% to my person (39 year old man). P: It is terrible, just terrible. Honestly, I must say, it is terrible to fill in. Because I don’t want to have anything to do with it any more. I: Do the questionnaire remind you of your epilepsy? P: Yes, it does, and the fact that it has been much worse, and it make me think; what if it gets worse again? (…) I really feel like they start to pull something up, and for some of the questions I think; why is it their concern? (67 year old woman). There are so many negative questions, so many around depression and suicide thoughts and that sort of stuff. It is very negative. And I think; what shall I do with that? Because I didn’t think I had those troubles, but when they are presented in that way in questionnaire, you actually come to be a bit negative yourself (32 year old man).
Lack of confidence in own ability to assess health status and need of contact	Who is to say that I’m right in my responses? (26 year old woman). Well, we are not supposed to be doctors, we are not supposed to assess our own health, because there are some people who are professionally educated to do that (39 year old man). It is hard to assess, because do I have a need to be contacted? Well, it is really difficult to assess, because I’m not the expert in this game (60 year old man). I thought to myself; well, is this really a proper reason for asking for contact. Because you do know that there is a need for cost-savings, so I wouldn’t want to intrude or be demanding. So, I feel that there must really be a proper reason, but you can be very insecure if the reason is proper enough, and that is hard. (26 year old woman).

### Supportive mechanisms

#### Increasing awareness of psychosocial problems

On account of PRO questions related to wellbeing and everyday life, patients who experienced the process favorably reported it as having a positive focus on psychosocial problems. These ‘soft’ questions were interpreted as a signal from the outpatient clinic that these were important areas for consideration and that the clinicians were interested in the whole person, not just the medical management. These patients thought of PRO-based follow-up as more personal and profound than the traditional follow-up, because previously the consultation focus was always limited to information about medicines and seizures. In addition, the comprehensive questionnaire and the weighting of the patient’s view and opinion on their own health status were understood as the clinicians taking patients more seriously. This awareness about potential psychosocial problems was valued by these patients and could potentially make it easier to handle psychosocial impact of epilepsy on everyday life.

#### Improving communication

PRO-based follow-up also resulted in an experience of improved communication for many patients. They stated that it provided them with a disease-specific vocabulary that allowed them to explain to the clinicians and to their relatives how they felt, and what it was like to live with epilepsy. They felt that the questionnaire prompted them to discuss psychosocial issues in relation to the disease with their relatives. These patients also found that the questionnaire ‘allowed’ them to initiate discussions with clinicians concerning problems like sadness, anxiety or problems with sexuality. Thus, PRO measures were perceived as a legitimization of conversations on a range of topics they might otherwise have avoided.

#### Increasing understanding of symptoms and disease

This group of patients felt that the PRO questionnaire could give rise to personal reflection. When reflecting on symptoms, they became more aware about the link between specific symptoms and the disease process, which resulted in a better understanding of the disease and its manifestations. Filling in the questionnaire made it easier for them to assess their need for contact, because PRO measures gave them a good understanding of their health status. The PRO questions could also clarify that epilepsy is an aspect in their everyday life and thus not restricted to seizures and medicine. In this manner, they reported that filling in the PRO questionnaire could lead to an increased acceptance and sense of security, because now they could legitimately relate their symptoms to epilepsy.

#### Facilitating change in health behavior

Filling in the PRO questionnaire further prompted some to take stock of their way of living with epilepsy. It made them pause to think carefully about the way in which their epilepsy was developing and reflect on their health-related actions. Thus, PRO measures gave rise to reflection and the reflection then led to acting differently when it came to sleeping patterns, dietary habits, medication management and alcohol intake. Many of the patients therefore felt it resulted in making different choices, choices that were more in line with recommendations from the outpatient clinics regarding health behavior and epilepsy. Thus, the PRO-based follow-up mediated processes by which some patients believed they were handling the epilepsy in a healthier way.

#### Strengthening autonomy, flexibility and freedom

Many patients valued assessing their own health status and need for contact, as it gave them a feeling of being in charge and a positive feeling of taking responsibility for their own disease. They appreciated the fact that they were given a choice as to whether or not they wanted a consultation. In this way, the PRO-based follow-up strengthened their autonomy, as it provided them with an actual influence on their follow-up. In addition, the fact that there were no routine consultations any more was highly valued, as it made follow-up more flexible. This flexibility had an impact on the patient’s everyday life, including work life. When they were no longer obligated to come to a routine visit, they reported that they felt less ill. They did not have to arrange to get off from work because they could fill in the questionnaire whenever it best suited them, and that gave them a sense of flexibility and freedom. The strengthened autonomy and the increased sense of flexibility and freedom signified that the epilepsy had to some extent faded into the background, and allowed their ‘patient identity’ to become less dominant.

### Inhibiting mechanisms

#### Inducing feelings of rejection and disconnection

A substantial barrier for PRO-based follow-up to support self-management for some of the patients in this study was an experience of feeling intercepted by the approach and therefore rejected by the health care system and the clinicians. For the most part, the experience associated with this rejection was lack of response. Even though these patients had stated problems in the PRO questionnaire, some reported that they had not received a response. Because questions were posed about wellbeing and problems such as with sexuality, they expected a response if they had indicated a problem in these areas. In such instances, they often found that the response given to their PRO data was overly superficial. As a result, they developed the clear conception that it was only those PRO measures that were related to seizures or medicine that the clinicians would respond to, and were thus convinced that different PRO questions rank differently. When these patients experienced no response to the problems they reported, their motivation for filling in the PRO measures decreased. Lack of response or the experience of a superficial response could leave such patients with a feeling of being abandoned and alone. In this way, they concluded that the PRO-based follow-up had intercepted their capacity to benefit from the expert and the experts’ assessment of the disease.

#### Incomprehension of the purpose of PRO-based follow-up

Another barrier some patients reported occurred in situations in which they missed or misunderstood the purpose of PRO-based follow-up. Some thought of the questionnaire strictly as the clinician’s work tool to collect data or as a cost saving tool for the healthcare system. They were not even aware that their PRO data were handled individually, and thought of the questionnaire as a survey for research purposes. Furthermore, when the questions made no sense, or patients could not see the link between the questions and the epilepsy, they wondered why the clinicians wanted information on all these areas.

#### PRO measures are too standardized and negative

Several patients pointed out that the PRO measures did not reflect the way their own epilepsy was manifest. In such situations, patients could not recognize themselves in the questionnaire. They found it too standardized and rigid and therefore demanded better possibilities to elaborate upon their answers. Thus, some of these patients found that the PRO measures restricted their communication about their epilepsy with the clinicians, as the questionnaire only allowed for patients to communicate on certain issues. In some instances, PRO measures were seen as a painful reminder of the severity of the disease. Certain questions in the PRO questionnaire, such as whether they had considered suicide, made some patients worry about what the future might bring. These patients emphasized that such questions were very negative, and explained that such questions negatively influenced their mood. Where they felt that the questions were particularly intrusive, some patients reported filling out the answers in a superficial manner in order to avoid feeling uncomfortable.

#### Lack of confidence in own ability to assess health status and need of contact

Some patients found it difficult to fill in the PRO questionnaire because they had a hard time distinguishing between symptoms related to epilepsy and more general symptoms or sensations. Where they lacked confidence in their own ability to assess their symptoms and health status, these patients emphasized that they had been given too much responsibility, and that made them feel insecure and unsafe. Several reported that they would prefer the clinicians doing the assessment. Furthermore, some found it difficult to assess their own need for contact, given that they did not see themselves as experts. Some also found it hard to request a contact if they had not stated any problems in the PRO measures. In this manner, patients had a clear sense of a PRO hierarchy, in that not all PRO questions legitimized a wish for contact. For the most part, questions related to seizures or medicine were viewed as more important than questions related to psychosocial problems.

### Sense of ownership

**Fig. 1:**
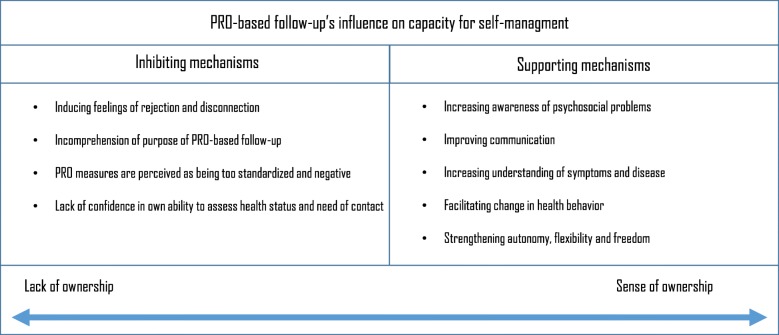
PRO-based follow-up’s influence on capacity for self-management. The figure represents inhibiting and supporting mechanisms for patients’ capacity for self-management. The double-arrow illustrates differences in experiences of ownership towards PRO-based follow-up on a spectrum from lack of ownership to sense of ownership as a possible explanatory mechanism

In searching for possible understandings of the conditions under which inhibiting and supporting mechanisms arose, we propose a sense of ownership towards PRO-based follow-up as a possible explanatory mechanism (Fig. 1). When we looked at cases in which PRO measures’ supporting mechanisms had been realized and in those in which the experiences were negative, we could see a difference in terms of the active agency of the patient in engaging with the system. Patients for whom the PRO-based follow-up seemed to increase their capacity for self-management were, for example, more likely to use possessive pronouns when they were referring to the PRO measures or the follow-up. Furthermore, these patients were more likely to express positive reflections regarding their role in the follow-up, and they described themselves as feeling comfortable with making the active choice regarding need for contact. Other patients, on the contrary, seemed to verbally distance themselves from PRO-based follow-up by emphasizing that it was the clinicians’ work tool, not their own. These patients furthermore tended to focus on what the healthcare system gained from PRO-based follow-up. In those cases, the patients tended to express experiences of PRO-based follow-up as a rigid and simplistic system, with no room for influence. Given that we interpret these differences in the active agency as related to differences in sense of ownership towards PRO-based follow-up, it seemed that ownership towards PRO-based follow-up created the conditions under which supporting mechanisms for self-management development could be realized.

## Discussion

In this interpretive description study we found signs of PRO-based follow-up leading to an increased capacity for self-management for some patients whereas, for other patients, PRO-based follow-up had no influence on their ability to manage the epilepsy or, in some instances had a negative influence on their perception of their follow-up and their contact to the clinic. For the most part, the supporting mechanisms for self-management found in our analysis reflect the general patterns that have been reported in the literature. For example, the improvement in communications is in line with findings reported in several other studies of PRO measures in clinical practice [7, 8, 11, 23]. The increased awareness of psychosocial problems and an increased understanding of the disease has also been recognized in previous research [7, 24, 25]. Accordingly, some of the inhibiting mechanisms we found have also been recognized in prior reports [7, 23]. The patients experienced that it was only PRO questions about seizures or medicines that the clinicians would respond to. This is in line with prior research in relation to the use of PRO measures in cancer care, which highlights that high score PRO data might not be explored further if health care providers do not relate them to the disease or the treatment [23]. Furthermore, there seems reason to consider if some of the inhibiting mechanisms found in our study are linked to elements of the PRO system not being designed to optimize patient or clinician engagement. Perhaps, one way to optimize clinician engagement could be to train clinicians in deliberate use of PRO measures in the patient-clinician encounter. Such a notion is in line with conclusions from research in oncology, pediatrics, and lung transplant, which argue that there is a need to train clinicians in how to use PRO measures and for recommendations on how to respond to issues identified by PRO measures [26, 27].

What stands out in our findings, compared to previous research in relation to PRO-based follow-up, are mechanisms related to patients’ experiences of being responsible for the assessment of health status and need of contact. In our study, it became evident that patients had different attitudes towards the responsibility and activity induced by PRO-based follow-up. This variation in preferences for being active is in agreement with Roter’s [28] framework of different models of patient-centered communications and the associated expected roles, and with Riiskjær’s [29] distinction between active and passive patients based on their information-seeking behavior. Furthermore, this variation in preferences for being active accords with the constructs behind the *Control Preferences Scale* (CPS) [30]. CPS is designed to elicit consumers’ preferences regarding participation in health-care decisions. Differentiating between different roles consumers can assume, the CPS offers an understanding of variations in the degree of control that different patients prefer in the context of treatment decision-making [30].

In the CPS, three roles are described -- the active role, the collaborative role and the passive role -- representing patient preferences for participating in decisions. With the CPS, patients are hypothesized to have different preferences about keeping control over treatment (the active role), sharing control with their physician (the collaborative role), or relinquishing control to their physician (the passive role) [30]. These typologies can be reflected in the patients’ experiences depicted in our study. In our findings, we saw both positive and negative attitudes towards the anticipated active patient participation that is embedded in PRO-based follow-up. For some patients, the embedded participation contributed to a strengthened autonomy and increased their sense of freedom, whereas for others, the embedded participation was accompanied with a feeling of rejection and disconnection to the clinicians. These negative attitudes can be interpreted as a preference for a passive role, whereas the positive experiences can be seen as preferences for a collaborative or active role in the follow-up. Thus, our findings give reason to consider if PRO-based follow-up will be most appropriate for patients who have an individual preference for holding an active or collaborative role in their follow-up.

There are some limitations to our study. Concerning transferability, we acknowledge that although patients with epilepsy are used as an exemplar case in our study, there may be some characteristics associated with this seizure disease that are dissimilar to the characteristics of other long-term conditions. Given that epilepsy is a condition in which fear of seizure can perhaps influence the patients need for ‘being close to’ the experts, further deliberation would be appropriate to determine whether the resistance towards the responsibility of assessing health status and need of contact identified in this population may be more extensive than with other patient populations. Another limitation to our study is the fact that the participants were all current users of the PRO-based follow-up. It could have been beneficial to also include patients who had dropped out of PRO-based follow-up. Thus, further research should both include other long-term conditions and attention to the group of patients that for some reason are not able to remain in PRO-based follow-up. Nonetheless, we expect these findings may have relevance for other contexts where patients with long-term conditions enter PRO-based follow-up, as they shed light on supporting and inhibiting mechanisms in PRO-based follow-up to support self-management.

In order to enhance the potential of PRO measures to increase patients’ capacity for self-management, we suggest bringing a clinical awareness to the issue of the quality of the introduction to the PRO-based follow-up to the patients. By enhancing comprehension of the explicit purpose of PRO-based follow-up, the patient’s sense of ownership towards the follow-up could potentially increase. Additionally, we suggest that the decision regarding the individual patient’s participation in PRO-based follow-up could be a shared decision (between clinician and patient) possibly supported by a decision aid. Such a decision aid could be based on the various possibilities and inhibitors found in this study. Given the issues that patients raised about the inappropriateness and negatively of some PRO measures, careful considerations regarding the selection of specific PRO measures are recommended before starting routine monitoring.

## Conclusion

This study draws attention to patients’ experiences of PRO-based follow-up as a means of self-management support. The findings demonstrate broad variation in the influences of PRO measures on patient’s self-management in life with epilepsy. Sense of ownership may explain this variation. We suggest supplementary clinical initiatives in order to enhance the benefits from PRO-based follow-up, particularly on how patients are allocated to this health care service.

## Additional file

Additional file 1: Epilepsy questionnaire. (PDF 1501 kb)

## Abbreviations

CPS: Control Preferences Scale
ID: Interpretive Description
PRO: Patient-reported outcomes
